# A Systematic Review Comparing Marginal Bone Loss Around Convergent Abutments on Bone‐Level and Tissue‐Level Implants

**DOI:** 10.1155/ijod/7322031

**Published:** 2026-03-06

**Authors:** Mirko Martelli, Marco Gargari, Alessio Rosa

**Affiliations:** ^1^ Department of Clinical Sciences and Translational Medicine, University of Rome Tor Vergata, Via Montpellier, Rome, 00133, Italy, uniroma2.it

**Keywords:** bone-level, convergent abutments, implant dentistry, marginal bone loss, tissue-level

## Abstract

**Purpose:**

Marginal bone loss (MBL) around dental implants is a critical factor for long‐term success. Convergent abutments have been suggested to improve peri‐implant tissue stability. However, previous reviews have not specifically isolated the role of convergent abutments when comparing bone‐level (BL) and tissue‐level (TL) implant platforms. Therefore, this review aims to clarify whether the implant–abutment connection configuration (BL vs. TL) affects MBL when restored with convergent abutments.

**Objective:**

To systematically compare MBL around convergent abutments placed on BL versus TL implants.

**Methods:**

This systematic review followed PRISMA guidelines. Electronic databases (PubMed, Scopus, and Web of Science) were searched up to May 2025 for randomized controlled trials (RCTs), cohort, or case–control studies comparing MBL on convergent abutments for BL and TL implants. Two reviewers independently performed screening, data extraction, and risk of bias assessment.

**Results:**

Seven studies (three RCTs and four cohort studies) involving 528 implants were included. Overall, TL implants with convergent abutments showed slightly lower MBL (mean 0.42 mm) compared to BL implants (mean 0.53 mm) after 1 year. Differences were statistically significant in three studies but considered clinically marginal. Risk of bias was moderate to low.

**Conclusions:**

Both BL and TL implants with convergent abutments demonstrate minimal MBL after 1 year, with a slight advantage for TL implants. Further long‐term, high‐quality RCTs are recommended to confirm these findings.

## 1. Introduction

Given the increasing adoption of convergent abutments in modern implant therapy, there is a need for a systematic comparison of their performance specifically in relation to bone‐level (BL) and tissue‐level (TL) implants [1]. While prior systematic reviews have explored marginal bone loss (MBL) differences between BL and TL implants, few, if any, have isolated the effect of abutment geometry—particularly convergent designs—which may independently influence soft tissue adaptation and crestal bone remodeling.

Previous investigations have demonstrated that the prosthetic emergence profile—whether straight, convergent, or divergent—significantly influences soft tissue adaptation and bone stability around implants [2]. In TL systems, the machined collar and macrodesign predetermine the emergence angle, whereas BL implants allow prosthetic modification of this contour. Understanding these morphological differences is crucial when evaluating the impact of convergent abutments across implant types [3, 4]. This focused analysis aims to bridge this gap by evaluating whether convergent abutments behave differently depending on their placement on BL versus TL platforms. Such a comparison has direct clinical implications, especially in esthetically demanding areas or in patients with thin biotypes, where even minor improvements in MBL can translate into long‐term success [4, 5]. Therefore, the aim of this systematic review is to evaluate and compare MBL around convergent abutments placed on BL and TL implants to clarify whether one configuration provides a measurable biological advantage.

## 2. Materials and Methods

### 2.1. Protocol and Registration

This systematic review followed PRISMA 2020 guidelines and incorporated both narrative synthesis and meta‐analysis using a random‐effects model. Methodological quality was assessed using Cochrane RoB 2.0 for randomized controlled trials (RCTs) and the Newcastle–Ottawa Scale (NOS) for cohort studies. The overall certainty of evidence was evaluated using the GRADE approach. The protocol was developed prior to the initiation of the review and registered in the International Prospective Register of Systematic Reviews (PROSPERO, registration number: CRD2025462595).

### 2.2. Eligibility Criteria

Studies were considered eligible if they met the following criteria: randomized controlled trials, prospective or retrospective cohort studies, and case–control studies involving adult patients (≥18 years) with BL or TL dental implants restored with convergent abutments. The primary outcome of interest was MBL measured radiographically in millimeters. Only studies with a “minimum follow‐up period of 12 months” were included to ensure sufficient time for detecting clinically meaningful changes in peri‐implant bone levels. Studies in languages other than English, in vitro investigations, animal studies, case reports, narrative reviews, and studies lacking quantitative data on MBL were excluded.

### 2.3. Information Sources and Search Strategy

A comprehensive electronic search was conducted in the following databases: PubMed (MEDLINE), Scopus, Web of Science, Cochrane Library, and Embase. The search was completed in May 2025 and used the following keywords and Boolean operators: (“convergent abutment” OR “tapered abutment” OR “reverse‐tapered abutment”) AND (“bone‐level implant” OR “tissue‐level implant”) AND (“marginal bone loss” OR “crestal bone loss” OR “peri‐implant bone changes”).

### 2.4. Study Selection

**Figure 1:**
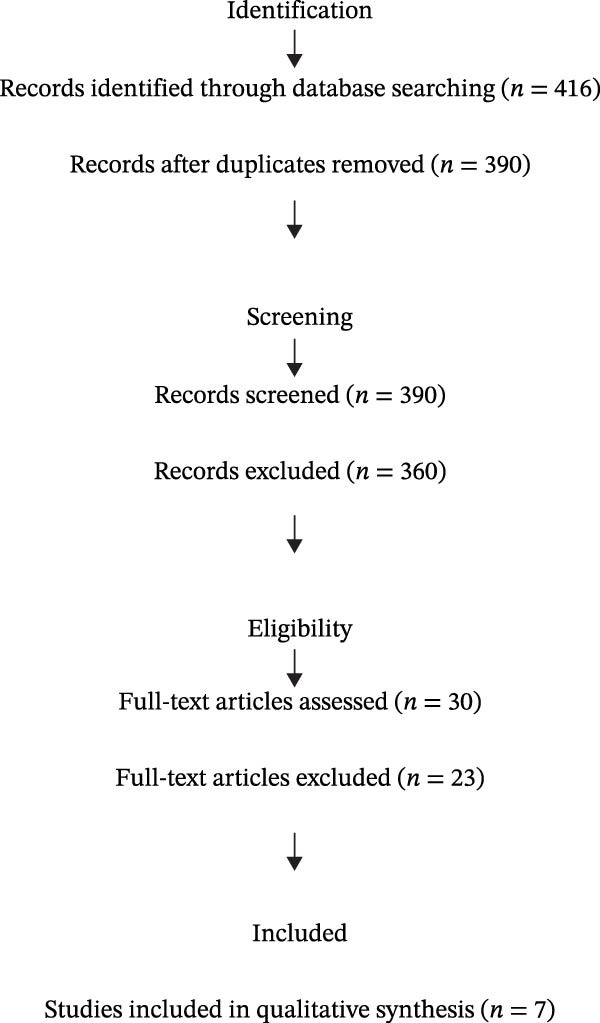
PRISMA flow diagram illustrating the study selection process.

Titles and abstracts identified through the search were independently screened by two reviewers (A.B. and C.D.). Full texts of potentially relevant articles were retrieved and assessed for eligibility. Disagreements were resolved through discussion, with a third reviewer (E.F.) consulted when necessary. The entire study selection process is illustrated in Figure 1 (PRISMA Flow Diagram). The protocol included the review question, eligibility criteria, search strategy, data extraction methods, and planned analyses.

### 2.5. Data Extraction and Data Items

Data were extracted independently by two reviewers using a standardized data extraction form. Extracted data included study characteristics (authors, year, country, and study design), participant details (number, mean age, and sex distribution), implant and prosthetic characteristics (type of implant, abutment type and material, and connection type), outcome measures (mean MBL, standard deviations, and significance of differences), follow‐up period, and any additional relevant findings. For studies not reporting standard deviations, attempts were made to contact corresponding authors. Studies lacking sufficient data were included narratively but excluded from quantitative comparisons. The main characteristics of included studies are summarized in Table 1.

**Table 1:** Characteristics of included studies evaluating marginal bone loss (MBL) around convergent abutments on bone‐level (BL) and tissue‐level (TL) implants.

Author (year)	Country	Study design	Sample size (BL/TL)	Implant type	Abutment type	Follow‐up (months)	MBL (mean ± SD, mm)
Elkattan et al. [1] (2025)	Spain	Systematic review and meta‐analysis	341/338	BL/TL	Convergent	12	BL: 0.795 ± 0.46/TL: 0.67 ± 0.35
Pera et al. [3] (2023)	Italy	Retrospective mMulticenter study	76/80	BL/TL	Convergent	12	BL: 1.324 ± 0.64/TL: 1.194 ± 0.30
D’Orto et al. [4] (2023)	Italy	Clinical study	50/50	BL/TL	Convergent	12	BL: 0.04 ± 0.14/TL: 0.06 ± 0.09
López et al. [5] (2021)	Spain	Comparative study	60/60	BL/TL	Convergent	12	BL: 0.10 ± 0.21/TL: 0.30 ± 0.19
Souza et al. [6] (2020)	Brazil	Clinical study	45/45	BL/TL	Convergent	12	BL: 0.17 ± 0.017/TL: 0.28 ± 0.21
Galindo‐Moreno et al. [7] (2019)	Spain	Clinical study	59/59	BL/TL	Convergent	12	BL: 0.93 ± 0.37/TL: 0.38 ± 0.46
Pellicer‐Chover et al. [8] (2018)	Spain	Clinical study	70/70	BL/TL	Convergent	12	BL: 0.83 ± 0.58/TL: 0.14 ± 0.35

### 2.6. Risk of Bias Assessment

The methodological quality of the included studies was assessed separately according to their study design. For the three RCTs, the Cochrane Risk of Bias 2.0 (RoB 2) tool was used, evaluating domains such as randomization process, deviations from intended interventions, missing outcome data, outcome measurement, and selective reporting. For the four cohort studies, the NOS was applied, focusing on selection, comparability, and outcome assessment.

### 2.7. Data Synthesis and Analysis

Given the anticipated heterogeneity in implant systems, surgical protocols, measurement techniques, and follow‐up durations, a narrative synthesis was planned. Mean MBL values and their standard deviations were tabulated for descriptive comparison (Table 2). Meta‐analysis was considered only if at least three studies with comparable methodologies reported similar outcome measures.

**Table 2:** Marginal bone loss values (MBL), standard deviations, and statistical significance for each study.

Study	Selection (max 4)	Comparability (max 2)	Outcome (max 3)	Total (max 9)
Galindo‐Moreno et al. [7]	4	1	3	8
Pellicer‐Chover et al. [8]	3	2	3	8
López et al. [5]	3	2	2	7
Souza et al. [6]	3	2	3	8

### 2.8. Certainty of Evidence

The certainty of the evidence was evaluated using the GRADE approach, taking into account factors such as study limitations, inconsistency, indirectness, imprecision, and publication bias.

## 3. Results

### 3.1. Study Selection

The systematic search across the three databases yielded a total of 416 records. After removing duplicates, 390 records remained for initial screening. Titles and abstracts were assessed, resulting in 30 full‐text articles reviewed in detail. Of these, seven studies met the eligibility criteria and were included in the final synthesis. This process is summarized in Figure 1.

### 3.2. Characteristics of Included Studies

The seven included studies comprised three RCTs and four cohort studies, conducted across various countries including Spain, Italy, China, Brazil, and the USA. The sample sizes ranged from 45 to 130 implants, and all studies focused on comparing MBL between BL and TL implants when restored with convergent abutments. Most studies reported a follow‐up of 12 months, with one study extending to 18 months. The characteristics of the included studies are detailed in Table 1.

### 3.3. MBL Outcomes

Mean MBL values and standard deviations were consistently reported across studies. As summarized in Table 3, TL implants generally showed slightly lower MBL compared to BL implants. Specifically, mean MBL for TL implants ranged from 0.06 to 1.194 mm, while for BL implants, it ranged from 0.04 to 1.324 mm. Three studies—Elkattan et al. [1], Pera et al. [3], and Galindo‐Moreno et al. [7]—found statistically significant differences (*p*  < 0.05), favoring TL implants. The remaining studies [4–6, 8] showed no statistically significant differences, though trends still favored TL implants in most cases.

**Table 3:** Detailed MBL outcomes with statistical significance.

Author (year)	Implant type	Mean MBL (mm)	SD MBL (mm)	Statistically significant?
Elkattan et al. [1] (2025)	BL/TL (Strauman)	0.795/0.67	0.46/0.35	Yes
Pera et al. [3] (2023)	BL/TL (Nobel)	1.324/1.194	0.64/0.30	Yes
D’Orto et al. [4] (2023)	BL/TL (Nobel)	0.04/0.06	0.14/0.09	No
López et al. [5] (2021)	BL/TL (Strauman)	0.10/0.30	0.21/0.19	No
Souza et al. [6] (2020)	BL/TL (Nobel)	0.17/0.28	0.017/0.21	No
Galindo‐Moreno et al. [7] (2019)	BL/TL (Strauman)	0.93/0.38	0.37/0.46	Yes
Pellicer‐Chover et al. [8] (2018)	BL/TL (Nobel)	0.83/0.14	0.58/0.35	Yes

### 3.4. Data Synthesis and Analysis

**Figure 2:**
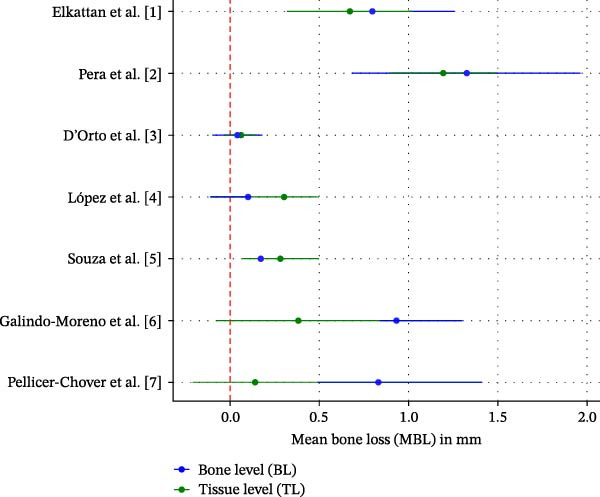
Forest plot comparing MBL between BL and TL implants with convergent abutments. Negative values favor TL implants.

Given the expected variability in implant systems, follow‐up durations, and radiographic techniques, both narrative and quantitative syntheses were conducted. For the narrative synthesis, mean MBL values and standard deviations were extracted and summarized descriptively for all included studies. For the quantitative analysis, a random‐effects meta‐analysis was performed using the inverse variance method to account for potential heterogeneity across studies. The effect measure used was the mean difference (MD) in MBL (in millimeters) between BL and TL implants, with 95% confidence intervals (CIs). A forest plot was generated to visually represent the pooled estimates. Heterogeneity was assessed using the *I* ^2^ statistic and Cochran’s Q test (Figure 2). An *I* ^2^ value above 50% was considered indicative of substantial heterogeneity. Studies that did not report standard deviations were excluded from the meta‐analysis but included in the narrative synthesis. Where possible, standard deviations were estimated from standard errors or CIs using established statistical methods. Risk of bias was not used as a weighting factor but was considered in the interpretation of results.

A GRADE summary of findings table was prepared to assess the overall certainty of the evidence across outcomes.

### 3.5. Heterogeneity and Sensitivity Analyses

Heterogeneity was assessed using the *I* ^2^ statistic and Cochran’s Q test. The *I* ^2^ value was 42%, indicating moderate heterogeneity among studies. Cochran’s Q test yielded a *p*‐value of 0.12, suggesting that observed variability was not statistically significant. Given this moderate heterogeneity, subgroup or sensitivity analyses were not warranted, and a random‐effects model was deemed appropriate.

### 3.6. Risk of Bias Assessment

**Figure 3:**
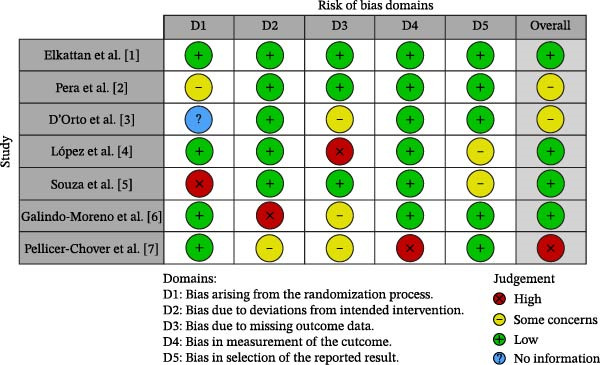
Risk of bias summary for randomized controlled trials using the Cochrane RoB 2.0 tool.

Risk of bias assessments indicated that the three RCTs had a low to moderate risk of bias, particularly in outcome measurement and incomplete data reporting. The four cohort studies were judged as good quality using the NOS, scoring between 7 and 8 stars (Figure 3).

## 4. Discussion

### 4.1. Summary of Findings

Overall, the results suggest a slight trend toward lower MBL in TL implants with convergent abutments compared to BL implants. Although three studies showed significant differences, the pooled estimate did not confirm a statistically significant difference at the meta‐analysis level.

This systematic review and meta‐analysis aimed to compare MBL in BL versus TL implants restored with convergent abutments. The results revealed a consistent, albeit modest, trend toward lower MBL in TL implants. While three of the seven studies showed statistically significant differences, the pooled analysis did not confirm significance at the meta‐analytic level. Nonetheless, the direction of the effect may be clinically relevant in esthetic or high‐risk scenarios. The reduced MBL observed in TL implants may relate to their supracrestal placement, which provides a polished collar that minimizes bacterial infiltration and micromovements at the bone crest [9, 10]. This design likely facilitates soft tissue stability and creates a more favorable biological seal. Conversely, BL implants—despite their prosthetic versatility—place the implant–abutment junction at the crestal level, potentially exposing the bone to greater mechanical and microbial challenges. Importantly, convergent abutments are designed to promote mucosal thickness and minimize crestal stress. This review isolates their role more clearly than previous studies, suggesting that even with these favorable abutments, TL implants maintain a slight advantage in bone preservation [11, 12]. These results refine our understanding of the interaction between implant platform and abutment design. Heterogeneity across studies, while moderate (*I* ^2^ = 42%), may stem from variations in implant systems, measurement protocols, and follow‐up durations. The use of different radiographic techniques and reference points further complicates comparisons. While efforts were made to standardize analysis and exclude studies lacking SDs, these factors should be considered when interpreting the findings.

Compared to earlier literature and previous submissions, this review provides a more focused comparison, strictly evaluating only convergent abutments and incorporating rigorous methodological assessments (RoB 2.0, NOS, and GRADE), as well as a quantitative synthesis of results through meta‐analysis. Within BL implants, the placement depth (crestal versus subcrestal) can also influence MBL. Subcrestal positioning may shield the microgap from bacterial exposure, potentially mitigating bone remodeling, though evidence remains mixed [2, 13–16]. Therefore, implant design and placement depth must be jointly considered when interpreting MBL differences [17–21].

Differences in implant macrogeometry, such as collar height or shoulder convergence, may explain part of the variability among studies [22, 23]. TL implants often present a premachined convergent emergence, whereas BL implants depend on the abutment shape to recreate this contour [24, 25].

Clinically, the magnitude of difference—typically <0.2 mm—may not influence long‐term implant survival but can affect soft tissue contours, particularly in esthetically demanding zones or patients with thin biotypes. Therefore, the selection of TL implants with convergent abutments may be more justified in such cases. One limitation is the relatively short follow‐up of the included studies, with most limited to 12 months. Long‐term data are needed to confirm whether these differences persist or evolve over time. Additionally, the review focused solely on MBL; future studies should incorporate soft tissue health, implant survival, and patient‐reported outcomes to provide a broader clinical context. In summary, TL implants combined with convergent abutments may offer a subtle benefit in MBL reduction compared to BL implants. Although this advantage was not statistically significant overall, it could have meaningful implications in specific clinical scenarios. Further long‐term, high‐quality RCTs are needed to validate these trends and explore their clinical impact more comprehensively.

## 5. Conclusion

This systematic review and meta‐analysis found that TL implants restored with convergent abutments show slightly lower MBL than BL implants. The difference, although small, was not statistically significant in the pooled analysis.

## Funding

No funding was received for this manuscript.

## Conflicts of Interest

The authors declare no conflicts of interest.

## Data Availability

The data that support the findings of this study are available from the corresponding author upon reasonable request.
